# Cordycepin Sensitizes Cholangiocarcinoma Cells to Be Killed by Natural Killer-92 (NK-92) Cells

**DOI:** 10.3390/molecules26195973

**Published:** 2021-10-01

**Authors:** Suthida Panwong, Methi Wathikthinnakon, Thida Kaewkod, Nunghathai Sawasdee, Yingmanee Tragoolpua, Pa-thai Yenchitsomanus, Aussara Panya

**Affiliations:** 1 Master of Science Program in Applied Microbiology (International Program), Faculty of Science, Chiang Mai University, Chiang Mai 50200, Thailand; suthida_panw@cmu.ac.th; 2 Department of Biology, Faculty of Science, Chiang Mai University, Chiang Mai 50200, Thailand; methi666@hotmail.com (M.W.); tda007suju@gmail.com (T.K.); yboony150@gmail.com (Y.T.); 3 Division of Molecular Medicine, Research Department, Faculty of Medicine Siriraj Hospital, Mahidol University, Bangkok 10700, Thailand; sawasdee111@gmail.com (N.S.); ptyench@gmail.com (P.-t.Y); 4 Siriraj Center of Research Excellence for Cancer Immunotherapy (SiCORE-CIT), Faculty of Medicine Siriraj Hospital, Mahidol University, Bangkok 10700, Thailand; 5 Research Center in Bioresources for Agriculture, Industry and Medicine, Faculty of Science, Chiang Mai University, Chiang Mai 50200, Thailand

**Keywords:** cordycepin, sensitization, immunomodulation, NK cell-based immunotherapy, cholangiocarcinoma, cancer treatment

## Abstract

Immunotherapy harnessing immune functions is a promising strategy for cancer treatment. Tumor sensitization is one approach to enhance tumor cell susceptibility to immune cell cytotoxicity that can be used in combination with immunotherapy to achieve therapeutic efficiency. Cordycepin, a bioactive compound that can be extracted from some *Cordyceps* spp. has been reported to effectively inhibit tumor growth, however, the mechanism of its tumor sensitization activity that enhances immune cell cytotoxicity is unknown. In the present study, we investigated the potency of cordycepin to sensitize a lethal cancer, cholangiocarcinoma (CCA), to natural killer (NK) cells. Treatment with cordycepin prior to and during co-culturing with NK-92 cells significantly increased cell death of KKU-213A as compared to solitary cordycepin or NK treatment. Moreover, sensitization activity was also observed in the combination of NK-92 cells and *Cordyceps militaris* extract that contained cordycepin as a major component. The cordycepin treatment remarkably caused an increase in TRAIL receptor (DR4 and DR5) expression in KKU-213A, suggesting the possible involvement of TRAIL signaling in KKU-213A sensitization to NK-92 cells. In conclusion, this is the first report on the sensitization activity of cordycepin on CCA cells to NK cytotoxicity, which supports that cordycepin can be further developed as an alternate immunomodulating agent.

## 1. Introduction

Cholangiocarcinomas (CCAs), or bile duct cancers, can be divided into three groups depending on the anatomical location of the tumor in the biliary tree, the locations of which are intrahepatic (iCCA), distal (dCCA), and perihilar (pCCA) [1]. The incidence rates of CCA show geographical variation, with a higher rate of occurrence in Eastern countries [2]. The highest incidence, with a rate of 85/100,000 population, is reported in northeastern Thailand [2]. CCA is a deadly cancer with a very poor prognosis due to the fact that most patients are diagnosed at an advanced, unresectable stage [3,4]. In advanced stages, the systemic chemotherapy has been used, however, the prolonged survival rate remains at less than 12 months since CCA commonly resists chemotherapy [5]. Thus, an alternative therapeutic approach is needed to combine with systemic chemotherapy, at least to prolong the survival of CCA patients.

Recently, immunotherapy approaches which stimulate the patients’ own immune cells have been developed to treat cancer [6]. The immune cells can be expanded *ex vivo* with or without genetic modification and infused back into the patients via adoptive cell therapy (ACT). Immunotherapy for the treatment of cancer has shown great efficacy in treating several diseases such as multiple myeloma and leukemia [7]. Natural killer (NK) cell plays important role in immune surveillance against tumors. It belongs to innate immunity and functions as the first line defense to rapidly eliminate the cancer cells without prior activation [8]. According to its critical anti-tumor activity, NK has been used as cellular immunotherapy with more than 100 clinical trial lists demonstrating the safety profiles [9]. For example, the NK cells-based adoptive cell therapy was recently used to treat refractory and relapsed acute myeloid leukemia in phase I clinical trial. The results showed neither dose-limiting toxicity nor significantly changes in numbers of other immune cells was reported [10]. Unlike the T lymphocytes, the NK cell recognized the cancer target via MHC independent mechanism; thus, it did not promote graft-versus-host responses. Apart from primary NK cells, the NK-92 cells have been established to allow continuously expansion of NK cells, but they retain several characteristics and capacities of the NK cells [11]. As natural NK cells, NK-92 cells do not require either prior activation or action in a MHC- dependent manner, which can avoid the consequences of graft-versus-host responses. Compared to primary NK cells, NK-92 cells are easy to expand *in vitro* and available as an off-the-shelf product that can be reproduced and lowers the cost of treatment [11]. Nowadays, NK-92 cells are approved by the US-FDA and reported to be used in a variety of cancers, including refractory blood cancers [12]. However, after infusion during immunotherapy there are several pathways by which cancer cells can escape and limit the effectiveness of the treatment [13]. The escape mechanisms potentially cause serious exhaustion of the immune cells, thus, a solution is urgently required to prevent resistance to immunotherapy and to enhance the activity of immune cells.

The immunomodulation effect is the ability to regulate the immune response by induction or suppression of immune activity [14]. Some agents which modulate the immune response, the so-called immunomodulation agents, have been proposed for use in cancer treatment because they modulate the anti-tumor activity of immune cells either by directly stimulating immune cell function or by indirectly sensitizing cancer cells to immune cell cytotoxicity. Previously, some herbal extracts and natural bioactive compounds has been reported to have immunomodulating properties [15,16,17]. Natural substances have been increasingly receiving attention because of the remarkable potential biological activity that they exhibit, and their high levels of safety since they have been used in traditional medicine. *Cordyceps* spp. has been used for centuries in Chinese medicine to promote health, and its activity to modulate the immune system has been recently reviewed emphasizing its probable utility in modern medicine [16,18]. Its bioactive compound, a nucleoside analogue (3′-deoxyadenosine) named cordycepin, exerts a broad range of pharmacological effects such as anti-oxidant, anti-inflammatory, anti-microbial, and anti-cancer activities [16,19]. The immunomodulatory activity of cordycepin has been reported to lower lipopolysaccharide-induced RAW 264.7 macrophage inflammation via decreasing the pro-inflammatory cytokine production involving TNF–α, IL-1β, IL-6 and inflammation-related proteins iNOS, and COX2 [20,21]. Interestingly, the effect of cordycepin- enriched *Cordyceps militaris* extract on promoting immunomodulation activity against breast cancer has also been recently reported [22]. It suppressed the regulatory T lymphocyte (Treg) population and up-regulates the cytotoxic CD8+ T lymphocytes, which caused a delay in breast cancer growth in mice [22]. This finding pointed to the considerable potential of *Cordyceps* extract for immune modulating activity. However, the tumor sensitization effect of cordycepin on promoting the anti-tumor activity of immune cells, as well as the underlying molecular mechanisms, have not yet been elucidated.

In this present study, we investigated the tumor sensitization effect of cordycepin and *C. militaris* extract, and their improvement of the anti-tumor activity of natural killer-92 (NK-92) cells against CCA. Our findings demonstrated that the treatment of cordycepin or *C. militaris* extract augmented the KKU-213A sensitivity to the NK-92 cell line resulting in a significant increase in cancer cell death.

## 2. Results

### 2.1. Cytotoxicity of Cordycepin in CCA Cell Lines KKU-213A, KKU-100, and KKU055

The cytotoxicity of cordycepin on CCA cells was first determined in three different CCA cell lines consisting of the well-differentiated KKU-213A, the poorly differentiated KKU-100, and the poorly differentiated KKU-055. Cell viability was measured after treatment with various concentrations of cordycepin for 24 h by using a cell viability assay which compared them to a no-treatment control (Figure S1). The half maximal cytotoxic concentration (CC_50_) was calculated and revealed that KKU-213A was the most sensitive to cordycepin since it had a CC_50_ of 119.1μM, followed by KKU-055 with a CC_50_ of 135.8 μM (Table 1). However, cordycepin treatment had no effect on the cell viability of KKU-100. Treatment with the highest tested concentration at 400 μM did not show any effect on the KKU100 survival rates, which potentially indicates that the CC_50_ of this cell might be much higher than 400 μM (Table 1).

### 2.2. The Killing Ability of NK-92 to Eradicate CCA Cell Lines

**Figure 1:**
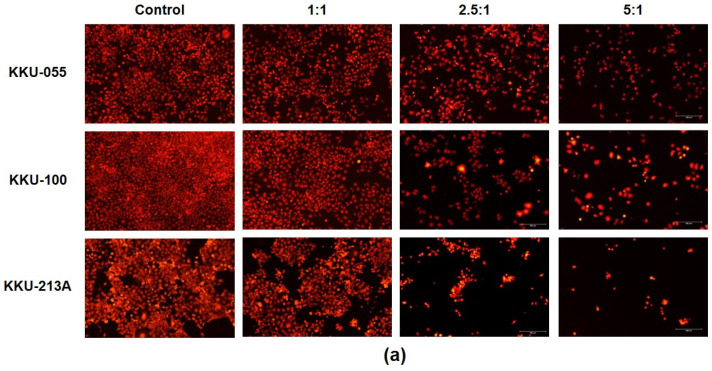

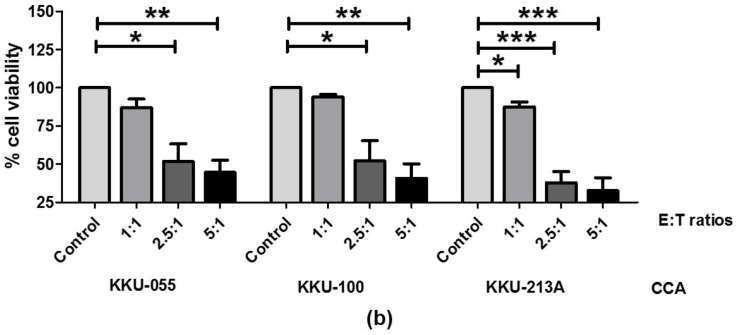
NK cell cytotoxicity in the killing of CCA cells. The NK killing ability was determined in KKU-213A, KKU-100, and KKU-055 by killing assay. The viable cells, after NK cell co-culture at E:T ratios of 1:1, 2.5:1, and 5:1, were observed under the fluorescence microscope (**a**). The fluorescence intensity was measured using the ImageJ program to represent the % cell viability relative to that of a no-treatment control (**b**) (* indicates *p* < 0.05; ** indicates *p* < 0.01 and, *** indicates *p* < 0.001.).

The activity of NK-92 cells to kill CCA cells was determined in KKU-055, KKU-100, and KKU-213A by using killing assays. The number of red fluorescence protein-expressing CCA cells was measured after 24 h of co-culturing with NK-92 cells at different effector-to-target ratios (E:T ratios). The fluorescence intensity was calculated to represent the number of living cells compared to a no-treatment control. The result showed that NK-92 cells had a greater ability to kill CCA cells in dose-dependent fashion (Figure 1a). At the highest E:T ratio of 5:1, it significantly decreased the cellular viability of KKU-055, KKU-100, and KKU-213A to 44.58 ± 7.94 %, 40.76 ± 9.44 %, and 32.70 ± 8.40 %, respectively (Figure 1b).

### 2.3. Treatment of Cordycepin Enhanced the NK-92 Killing Ability

**Figure 2:**
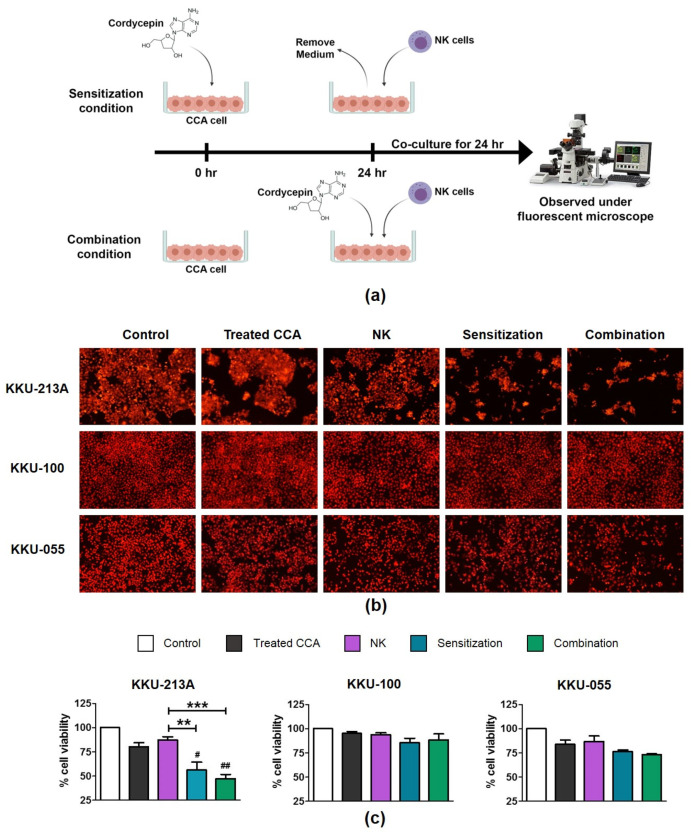
Effect of cordycepin on enhancing NK-92 cytotoxicity to kill CCA cells. The scheme represents the testing conditions used to investigate the cordycepin effect on enhancing NK cell cytotoxicity (**a**). The effect of cordycepin at a concentration of 50 µM to enhance NK killing ability was determined in KKU-213, KKU-100, and KKU-055, by killing assay. The killing assay was conducted with 24-hour incubation time using the E/T ratio of 1:1. The number of living cells after NK cell co-culturing under sensitization and combination conditions was observed under the fluorescence microscope (**b**). The fluorescence-intensity was measured and represented as %cell viability compared to a no-treatment control set at 100% (**c**)**.** The statistical differences were analyzed comparing combined cordycepin-NK treatment vs. single NK treatment, in which ** indicates *p* < 0.01; and *** indicates *p* < 0.001, whereas the statistical differences between combined cordycepin-NK treatment and single cordycepin treatment are designated as # indicates *p* < 0.05; and, ## indicates *p* < 0.01.

The immunomodulation activity of cordycepin on enhancing NK killing function against CCA cells was determined. Tests under two different sets of conditions were performed to discern the effects of cordycepin. They were either: (1), under sensitization conditions, in which CCA was pre-treated with cordycepin before co-culturing with NK-92 cells; or (2), under combination conditions, in which cordycepin was treated during co-culturing with NK-92 cells (Figure 2a). The number of living cells remaining was measured after a 24-hour co-culturing and it was revealed that the cordycepin effect was to improve NK-killing activity.

At E/T ratio of 1:1, the treatment of low-toxicity-dose cordycepin (at 50 μM) combined with NK-92 cells, increased KKU-213A cell death under either sensitization or combination conditions (Figure 2b). It caused the reduction of KKU-213A cell viability to 56.20 ± 8.21% under sensitization conditions, and to 47.23 ± 4.47% under combination conditions compared to 80.39 ± 4.10% and 87.43 ± 3.27% in cordycepin and NK solitary treatments, respectively (Figure 2c). Furthermore, treatment with the higher concentration of cordycepin at 100 μM caused more cell death under both conditions, which therefore reduced cell viability to 43.09 ± 6.78% and 37.10 ± 11.47% under sensitization and combination conditions, respectively (Figure S2). Cordycepin treatment caused only slight changes in KKU-055 (Figure 2b). The cell viability in combination treatment was 73.15 ± 0.87%, compared to 84.07 ± 4.10% in cordycepin single treatment, and 86.82 ± 5.71% in NK-92 single treatment (Figure 2c) where the pre-treatment of cordycepin caused no significant changes under sensitization conditions. In KKU-100, which was the most resistant cell to cordycepin, cordycepin caused no changes in cell viability under both sensitization and combination conditions (Figure 2b,c).

### 2.4. The Effects of Cordycepin on Modulating CD95, DR4, DR5, MICA/B Expression in CCA Cell Lines

Treatment of cordycepin was shown to be effective in enhancing NK killing activity of KKU-213A cells. We thus attempted to investigate the mechanism underlying the action of cordycepin on sensitization of cancer cells to NK cells. Previously, the association of death receptor upregulation (i.e., TRAIL receptor/DR, Fas receptor/CD95) and immune cell response has been reported to contribute on the anti-tumor activity of the immune cells and immune modulating activity [23,24]. 

**Figure 3:**
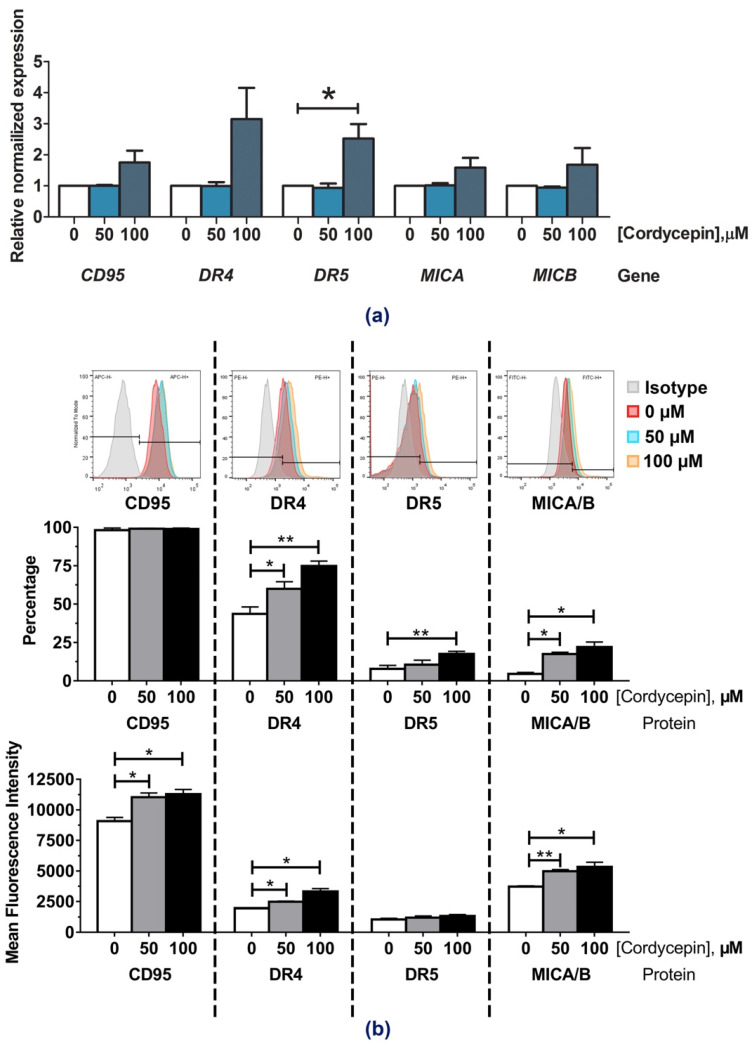
The effects of cordycepin on modulating CD95, DR4, DR5, MICA/B expression. Cordycepin at concentrations of 0, 50 and 100 µM was put in with KKU-213A cells for 24 h to determine its effect on CD95, DR4, DR5, MICA/B expression-alteration in either mRNA (**a**) or protein levels (**b**) by using real-time PCR and flow cytometry respectively. (* indicates *p* < 0.05, and, ** indicates *p* < 0.01.).

In addition, the involvement of MICA/B activation proteins was recently reported to impact on anti-tumor activity of NK cell [25]. Therefore, the extent of CD95, DR4, DR5, MICA/B expression alterations were then determined after cordycepin treatment for 24 h in either mRNA or protein levels. The real-time PCR demonstrated that cordycepin treatment at a concentration of 100 µM up-regulated the mRNA expression of *DR4* and *DR5*, in addition to slightly increased the expression of *CD95* and *MICA/B* in KKU-213A (Figure 3a). Concordantly, flow cytometry confirmed an increase of DR4 and DR5 positive cells and their corresponding mean fluorescence intensities in KKU-213A (Figure 3b). Interestingly, the mean fluorescence intensities of CD95 and MICA/B were also increased after treatment with cordycepin (Figure 3b). However, the treatment caused no changes to those genes in KKU-100 and KKU-055 (Figure S3).

### 2.5. The Effects of Cordycepin on NK-92 Cell Proliferation

**Figure 4:**
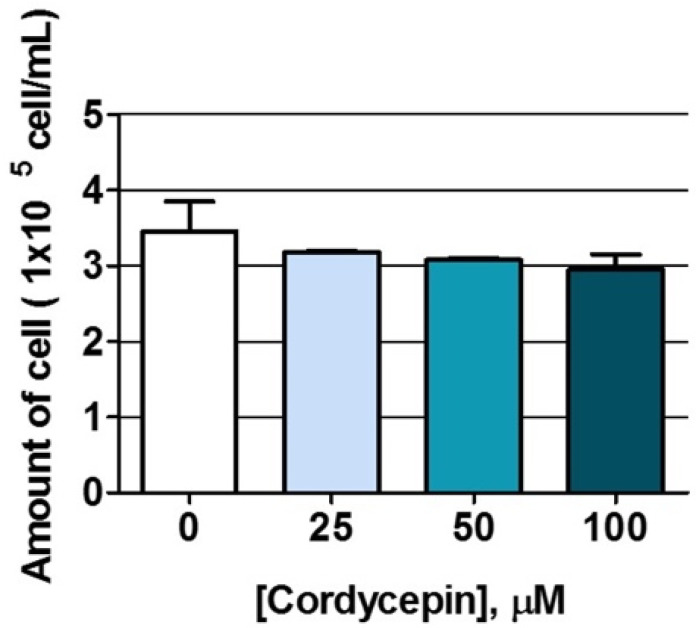
The effects of cordycepin on NK cell proliferation. The amount of NK cells was examined after a 24-hour cordycepin treatment at concentrations of 0, 25, 50, and 100 µM using trypan blue exclusion assay.

To determine the effect of cordycepin on NK cells, we measured NK cell proliferation after cordycepin treatment by using trypan blue exclusion assay. The cells were harvested after a 24-hour treatment of cordycepin at concentrations of 0, 25, 50, and 100 µM. The result showed that there were approximately 3 × 10^5^ cells/mL in the no-treatment control (Figure 4). However, no significant changes of NK cell numbers were observed at all concentrations of cordycepin treatment.

### 2.6. Cordyceps militaris Extract Combined with Cordycepin Enhanced Cytotoxicity of NK-92 to Eradicate CCA Cell Lines

Cordycepin is a bioactive compound present in some *Cordyceps* spp. To investigate the biological activity of those *Cordyceps* spp. containing cordycepin on enhancing NK cytotoxicity, the immunomodulation activity of *Cordyceps militaris* and *Isaria tenuipes* extracts were compared. The fruiting body and substrates of mushrooms *C. militaris* and *I. tenuipes* were extracted with water (1:10, *g/v*), and the cordycepin was analyzed via high performance liquid chromatography (HPLC). As expected, a peak level of cordycepin compared to the standard level of cordycepin was observed in *C. militaris* extract, but not in *I. tenuipes* extract (Figure S4).

**Figure 5:**
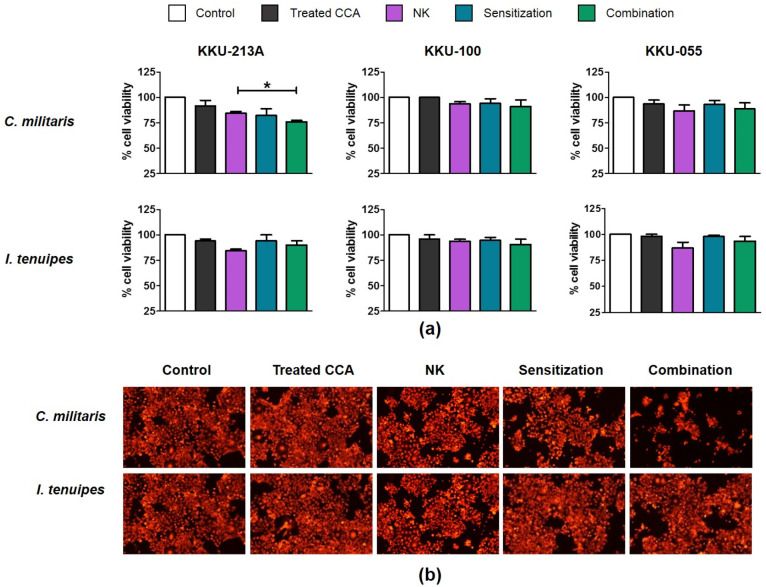
The NK cell activity with the extracts enhancement to eliminate CCA cell lines. The effect of *C. militaris* and *I. tenuipes* extracts to enhance NK killing ability was determined in KKU-213A, KKU-100, and KKU-055 by killing assay (**a**)**.** The killing assay was conducted with 24-hour incubation time using the E/T ratio of 1:1. The number of KKU-213A living cells after a combined NK cell co-culture under sensitization and combination conditions was observed under the fluorescence microscope (**b**)**.** The statistical differences were analyzed comparing combined cordycepin-NK treatment vs. single NK treatment, in which * indicates *p* < 0.05.

The cytotoxicity of these extracts to CCA cell lines was determined by using the cell viability assay to select for sub-lethal extract dosages. Our results showed that treatment with high concentration of *C. militaris* extract caused a reduction in cell viability whereas the *I. tenuipes* extract treatment had no effect on cell viability, even at the highest test concentrations (400 µg/mL) (Table 2). The CC_50_ values of *C. militaris* extract on KKU-213A, KKU-100, and KKU-055 were 85.48, 122.70, and 88.88 µg/mL, respectively (Table 2). The killing assay was then performed to determine the activity of *C. militaris* and *I. tenuipes* extracts on modulating NK cells. Treatment of *C. militaris* with a sub-lethal dose (31.25 µg/mL) enhanced NK-92 killing activity in KKU-213A under both sensitization and combination conditions (Figure 5a,b). At an E:T ratio of 1:1, it caused the reduction of KKU-213A cell viability to 82.27 ± 6.60% and 76.13 ± 1.31% under sensitization and combination conditions compared to the no-treatment control (84.41 ± 1.62%) (Figure 5a). The treatment tended to enhance NK-92 ability to kill KKU-055 but caused only slight effects on that of KKU-100 (Figure 5a). Notably, the *I. tenuipes* extracts did not affect the NK activity and showed no significant changes to living cell numbers under the fluorescence microscope (Figure 5b).

## 3. Discussion

Immunotherapy is a novel therapeutic approach that guides the way forward for alternative treatments, especially for cancers that currently have no effective curative treatments. CCA is exactly the kind of cancer for which standard treatments fail to prolong survival rates. The 5-year survival rate for those who are not able to have surgery is less than 12-months [5]. Thus, the research on an immunotherapeutic approach is necessary to improve patient survival rates and quality of life.

The NK cells, a member of the innate immune system, play a role as the first line of defense in cancer immunosurveillance. In this present study, we investigated the efficacy of NK cell immunotherapy in eradicating certain CCA cell lines *in vitro* and demonstrated a way to enhance NK efficacy by combining it with cordycepin as an immunomodulatory agent. In our study, NK-92 cells showed anti-tumor activity which killed several different types of CCA cell lines including KKU-213A, KKU-100, and KKU-055. However, the magnitude of NK-92 cytotoxicity was varied among these three different cell types. The most effective result was observed in KKU-213A in which NK-92 cells caused more than 67.30 ± 12.29% of cell death at an E:T ratio of 5:1, whereas KKU-055 was more sensitive to NK-92 cells than KKU-100 was (Figure 1). Since KKU-213A, KKU-100, and KKU-055 were sourced from different patients in different stages of the disease, the genetic backgrounds and corresponding expression profiles might be possible factors that contributed to such varied NK-92 activity. KKU-213A was sourced from a 58-year-old male donor and characterized as well-differentiated intrahepatic CCA with high invasiveness and aggressiveness [26]. KKU-100 exhibits the features of poorly-differentiated hilar CCA and was sourced from a 65-year-old female donor with less invasive CCA than that of the KKU-213A [27]. The other, the poorly differentiated KKU-055, was sourced from a 56-year-old male donor [28]. Interestingly, the substantial differences of gene expression levels were previously reported among these CCAs, and they have been proposed as the major cause of unsuccessful CCA therapy [29]. In addition, the natural differences of CCA cell lines might impact not only the NK cytotoxicity-sensitivity but also affect their sensitivity to cordycepin treatment. In our study, KKU-213A had the highest sensitivity to cordycepin and *C. militaris* extract with a CC_50_ of 119.10 µM and 85.48 µg/mL, respectively (Table 1 and Table 2). Concordantly, the immunomodulation activity of cordycepin on enhancing the NK-92 cell cytotoxicity was more pronounced in KKU-213A, which was the most sensitive type of cell (Figure 2 and Figure 5). Cordycepin treatment showed enhancement of NK-92 killing activity against KKU213A and KKU-055 but caused only slight effects on KKU-100. The most potential activity was found in KKU-213A in which NK-92 at the E:T ratio of 1:1 killed more than 43.80 ± 8.21% and 52.77 ± 4.47% when treated within the parameters of the sensitization and combination models respectively (Figure 2b,c).

The mechanism underlying cordycepin effect on enhancing NK-92 function was investigated and divided into two directions of analytical inquiry. First, the effect of cordycepin on cancer sensitization was investigated by monitoring the changes of NK-related gene expression levels after cordycepin treatment. The expression-alteration of death receptors included CD95, DR4, DR5 and activation ligands such as MICA and MICB were monitored. Treatment of cordycepin at 100 µM showed an increase in the mRNA expression levels of *DR4* and *DR5* (Figure 3a) which were concordant with the elevated protein expressions reflected by the higher number of DR4 and DR5 positive cells and mean fluorescence intensity (Figure 3b), whereas cordycepin treatment only caused an increase in the CD95 and MICA/B mean florescence intensity but did not change the percentage of positive cells. The result suggested TRAIL signaling involvement in the cordycepin mechanism, and that Fas signaling, and MICA/B might partially contribute to these actions as well. The DR4 (TRAILR-1) and DR5 (TRAILR-2) are N-glycosylated and O-glycosylated antagonistic receptors which can be found on cancer cells. Both receptors are closely related and share 58% of an external domain and 65% of an intracellular domain, and can bind with TRAIL (Apo2 ligand), which is an effector ligand on NK cells [30]. The DR4/DR5 and TRAIL complex activates programmed cell-death signaling by stimulating the FAS-associated death domain protein (FADD) and the caspase-8 proenzyme, leading to apoptosis and tumor cell death [31]. On the other hand, CD95 (Fas/APO-1/TNFRSF6) is a member of the class of death receptors which can be expressed on cancer cells. The CD95 receptor can interact with CD95L (CD178/TNFSF6), which is expressed on T lymphocytes and NK cells to stimulate pro-apoptotic factors such as caspase-8 resulting in cancer cell apoptosis [32]. The major histocompatibility complex class I chain related-protein A (MICA) and B (MICB) act as activation ligands, which are plentifully expressed on various carcinomas and can interact with natural killer group 2D receptors (NKG2D), thus triggering the cytolytic responses of NK cells and eventuating the eradication of tumors [33].

Previously, cordycepin has been reported as able to increase cancer cell death when combined with other treatments. The reported cordycepin signaling pathway in other cancer cells was revealed through regulating multiple pathways that included AMPK/mTORC1 [34,35], caspase [36], and c-Jun N-terminal kinase (JNK) signaling [37,38]. Cordycepin, in combination with gemcitabine or 5-fluorouracil (5-FU), was shown to improve chemosensitivity in gallbladder cancer GBC-SD cells by inhibiting mTOR complex 1 (mTORC1) and by promoting multiple drug resistance (MDR) degradation via activated AMP-activated protein kinase (AMPK) signaling [39]. Cordycepin was also found to sensitize human hepatocellular carcinoma (Hep3B) cells to TRAIL-mediated apoptosis by co-treating with TRAIL, which leaded to pro-apoptosis up-regulation and anti-apoptosis protein down-regulation via decreasing JNK phosphorylation [40]. Importantly, cordycepin mechanisms in CCA apoptosis have been demonstrated to promote QBC939 and RBE cell death via regulating alteration of caspase-3, Bcl-2, and Bax protein expression [41]. Furthermore, cordycepin caused apoptosis in HuCCT1 cells by inhibiting DEK proteins and decreasing ERK1/2 signaling [42]. However, there is no conclusive evidence of those reported cordycepin mechanisms being involved in the immunomodulation activity of cordycepin on CCA sensitization.

As far as the other direction is concerned, the direct effect of cordycepin on NK cell proliferation was examined based on the outcome of the combination treatment in which cordycepin improved NK cytotoxicity when administered to the cell concurrently with NK cells. The result demonstrated that cordycepin had no effect on NK cell viability; nevertheless, cordycepin also had no effect on NK cell multiplication after 24 h of treatment (Figure 4). Indeed, cordycepin might contribute to modulating NK activity either in terms of quantity or of quality. Our experiment demonstrated that cordycepin was not involved in quantity control; however, we cannot exclude the possibility that cordycepin modulates the quality of NK cell. To prove the hypothesis, we investigated the effect of cordycepin on altering the mRNA expression level of interferon gamma (IFN–γ) and granzyme cytotoxic protein by using real-time PCR. However, the direct treatment of cordycepin to NK cells showed no significant changes on IFN–γ and granzyme expression (Figure S5) reflecting the cordycepin might not directly affect to NK function. On the other hand, the indirect effect of cordycepin activating NK cells by modulating the cytokine profile from cancer cells and in turn controlling NK cell function would be one of several possible mechanisms. Further investigation of the cordycepin mechanism is needed to explore the immunomodulation mechanism of cordycepin which could be performed through investigating the changes of pan-expression levels of either mRNA or proteins using transcriptomics or proteomics, respectively.

The toxicity and safety of cordycepin in humans during therapeutic application needs to be taken into consideration. Cordycepin content has been profiled as the major bioactive compound in *Cordyceps* species, which are well known for their use in traditional Chinese medicine. Cordycepin content was reported to be approximately 0.97% and 0.36% in the fruiting body and corpus extracts of *C. militaris*, respectively [43]. In our experiment, the immunomodulation of cordycepin acquired the minimum concentration of 50 µM (12.5 µg/mL) which theoretically required 1.30 mg/mL of the fruiting body-part extract or 3.49 mg/mL of the corpus-part extract to achieve 12.5 µg/mL of cordycepin. Interestingly, we demonstrated that *C. militaris* extract at the concentration of 31.25 µg/mL showed that it could improve NK cytotoxicity–especially in the most sensitive cell line, the KKU-213A (Figure 5a,b). The result suggested that cordycepin in combination with other bioactive substances in the extract was able to synergistically promote the NK function and could reduce the effective dose of *C. militaris* extract from the estimated concentrations which could minimize concerns of potential toxicity from high doses of the extract.

The therapeutic applications of cordycepin have been reported from the perspective of several physiological processes, including those incorporating mRNA polyadenylation inhibitory [44], anti-inflammatory [45], anti-tumor [19], and anti-cancer properties [46]. Moreover, cordycepin is a great option to utilize instead of traditional chemotherapy drugs due to it being a natural compound possessing less unpleasant side effects than synthetic drugs. However, there are certain limitations that should be placed on cordycepin treatment suggested by our findings, since the effectiveness of cordycepin depends on cell types and under which conditions they exist. Furthermore, high cordycepin concentrations have been reported to cause some serious side effects, such as eryptosis, or liver and kidney damage [47]. Therefore, the effective doses for certain diseases have to be considered to be lower than those of toxic doses. Cordycepin dosages have been reported in both *in vitro* and *in vivo* studies. In animal models, the various doses of cordycepin have been studied. For example: the doses ranging from 2.0–88.9 mg/mL were tested in rats, 0.5–72.0 mg/mL in mice, and 140 mg/mL in hamsters [48]. Recently, a phase 1 clinical trial (ClinicalTrials.gov Identifier: NCT00003005) of cordycepin plus pentostatin chemotherapy, treating patients with refractory acute lymphocytic or chronic myelogenous leukemia, was completed; and there is also an ongoing phase 2 clinical trial [49]. In the phase 1 clinical trial, a formula consisting of cordycepin at the maximum dosages of 48 mg/m^2^, in combination with pentostatin, was tested on humans aged 18 years [50]. Therapy with 48 mg/m^2^ (mg/kg) of cordycepin concentration was approximately 96 mg in reference to the average total adult (18 years and older) body surface area (BSA) of around 2.00 m^2^ [51]. Notably, our study showed that immunomodulation activity of cordycepin was observed at the concentration of 50 µM (12.5 µg/mL or 12.5 mg/kg) and 100 µM (25 µg/mL or 25 mg/kg) suggesting that it has considerable potential to be safely used as an immunomodulatory agent with minimal adverse side effects.

In summary, our study showed that cordycepin, which is a natural bioactive compound, potentially enhances the NK-92 cell ability to eliminate CCA cancer cells, especially when applied to the well-differentiated intra-hepatic KKU-213A cell line. The increase in TRAILR (DR4 and DR5) expression might at least partially contribute to cordycepin immunomodulation activity through cancer cell sensitization. However, the actual mechanism of how cordycepin promotes NK cell ability needs to be further studied to fill the knowledge-gap on cordycepin effect on NK cells. Our findings have supported the hypothesis that cordycepin can be further developed as an alternate immunomodulating drug for NK cell adoptive treatments.

## 4. Materials and Methods

### 4.1. Cell Culture

Human CCA cell lines, KKU-213A, KKU-100 and KKU-055 were a kind gift from the Faculty of Medicine, Siriraj Hospital, Mahidol University, Thailand. The cells were purchased from the Japanese Collection of Research Bioresources Cell Bank (JCRB, National Institute of Biomedical Innovation, Osaka, Japan). KKU-213A, KKU-100 and KKU-055 were originally established from primary tumors of Thai CCA patients in which KKU-213A [26] was described as poorly differentiated squamous cell carcinoma whereas KKU-100 and KKU-055 was characterized as poorly differentiated adenocarcinoma [52]. The cells were cultured in Gibco Dulbecco’s Modified Eagle Medium: Nutrient Mixture F-12 (DMEM/F-12) (Thermo Fisher Scientific, Waltham, MA, USA) supplemented with 10% fetal bovine serum (Thermo Fisher Scientific) and incubated 37 °C, 5% CO_2_ incubator.

### 4.2. Cytotoxicity of Cordycepin on CCA Cell Lines

The toxicity of cordycepin to CCA cell lines was measured by using PrestoBlue™ reagent (Invitrogen, Carlsbad, CA, USA). The CCA cell lines were cultured in 96-well plates with approximately 1 × 10^4^ cells per well with a 2-fold serial dilution of cordycepin (Sigma-Aldrich, Darmstadt, Germany) between 3.125–400 µM, and incubated for 24 h. Then, the culture media was replaced with the PrestoBlue™ reagent (Invitrogen) at 100 µL per well. The reagent has the active ingredient called resazurin, which can be reduced by receiving electrons from the products of cellular respiration in living cells i.e., NADPH, FADH, FMNH, NADH and cytochromes. The reaction causes the change of resazurin to resorufin which turns the color from blue to pink which can be measured by recording the changes of absorbance at 570 nm (using 600 nm as a reference wavelength). The magnitude of color changes depends on the metabolic activity in living cells.

### 4.3. Killing Assay

To investigate the effect of cordycepin on enhancing NK cell activation for CCA elimination, the killing assay was performed. Briefly, the mCherry-expressing CCA cell lines (fluorescent cells) were seeded in 96-well plates at approximately 7 × 10^3^ cells per well and incubated for 24 h. The cytotoxicity of NK cell to kill CCA was measured after co-culturing the NK (effector cells) and CCA (target cells) in the different proportion of effector cells to target cells. The desired proportion was described as effector to target ratio (E/T ratio). In the combination condition, the culture media was replaced with a combination of NK cells at E/T ratios of 1:1, 2.5:1, and 5:1, with cordycepin (Sigma-Aldrich) at concentrations of 50 and 100 µM/mL. In the sensitization condition, the CCA cells were plated with cordycepin at concentrations of 50 and 100 µM/mL, in 96-well plates for 24 h. Later, the culture media was replaced with NK cells at E/T ratios of 1:1, 2.5:1, and 5:1. At 24 h after co-culturing of NK and CCA cells, the living CCA cells which remained on the plate were observed under fluorescent microscope (Eclipse Ts2R-FL, Nikon, Tokyo, Japan) and analyzed for red fluorescence intensity using ImageJ software. Briefly, the living CCA cells from different conditions were photographed with the identical exposure times and objective lens (100x). Three independent images from each condition were input to the ImageJ software. Entire areas in the images were selected and used for measuring mean fluorescence intensity (MFI) by using “Analyze and Measure” mode to determine the average number of pixel intensity of the image (Figure S6). The MFI values from the software were compared and used to calculate the percentage of cell viability relative to that of the non-treatment control which set as 100% as the following equation:

$$\%\;Cell\;viability\;=\;\left[\left(mean\;fluorescence\;intensity^{TEST}/\;mean\;fluorescence\;intensity^{CONTROL}\right)\;\times \;100\right]$$

The values from three independent experiments were analyzed for statistical differences.

### 4.4. Quantitative Real-Time Reverse Transcription PCR (qRT-PCR)

Determination of cordycepin effects on gene expression levels of CCA cell lines by RT-PCR. The cells were plated at approximately 1 × 10^5^ cells per well, together with the cordycepin at concentrations of 25, 50, and 100 µM, in 6-well plates and then incubated for 24 h. Next, the cells were collected in Trizol^TM^ reagent (Invitrogen, Carlsbad, CA, USA). Then, the cells’ RNA was extracted, and its’ quality was measured using nanodrop. Then the RNA was converted into cDNA using the cDNA synthesis kit (Meridian Bioscience, Cincinnati, OH, USA). The 0.5 µg of templates, the primers specific to *CD95*, *DR4*, *DR5*, *MICA*, *MICB* (Table S1), and 2x SensiFAST SYBR No-ROX Mix (Meridian Bioscience, Cincinnati, OH, USA were placed in an iCycler Thermal Cycler (Bio-Rad Laboratories, Hercules, CA, USA). The data were calibrated employing house-keeping genes (GAPDH) and fold changes calculated and compared to a relative value of 1.0 representing the condition set lacking cordycepin.

The effect of cordycepin on gene expression levels in NK cells was determined by Quantitative real-time reverse transcription PCR (qRT-PCR). The NK cells were treated with 50, and 100 µM for 24 h. The RNA isolation was performed using Trizol^TM^ reagent (Invitrogen), and the RNA quality was measured using nanodrop. After that, the RNA was converted to cDNA by using a cDNA synthesis kit (Meridian Bioscience, Cincinnati, OH, USA). The gene expression levels of *INF-γ, granzyme* genes were determined using the specific primers (Table S1) as described above.

### 4.5. Trypan Blue Exclusion

To investigate the effect of cordycepin on NK cell proliferation, the trypan blue staining was conducted. Briefly, the NK cells were plated in 6-well plates at approximately 2 × 10^5^ cells per well in the absence of presence of cordycepin at the concentrations of 25, 50, and 100 µM. After 24 h of incubation, the living NK cells were stained with trypan blue and counted under the light microscope.

### 4.6. Plant Extraction

Dried fruiting bodies of *Cordyceps militaris* and *Isaria tenuipes* were obtained from AAVA Group Co., Ltd. (Bangkok, Thailand). Briefly, the sample was ground and soaked in sterile distilled water at a ratio of 1:10 (*w/v*), followed by maceration with distilled water at 45 °C for 3 more hours. The extract was then filtered before evaporating via a rotary evaporator, at 45 °C, under a reduced pressure of 50 mbar. The extracts were then dried by lyophilisation and kept at -20 °C until used.

### 4.7. High Performance Liquid Chromatography (HPLC) of the Extracts

The cordycepin was determined in the extracts by using High Performance Liquid Chromatography (HPLC). A reference cordycepin sample (Sigma-Aldich, Burlington, MA, USA) was used as the standard from which to determine the quantities of cordycepin in the extracts of *C. militaris* and *I. Tenuipes,* which were prepared at concentrations of 10 mg/mL. 50 µL of cordycepin and extracts that had been filtrated with a 0.45 µM micro-filter were injected into the HPLC system (Agilent Technologies, Santa Clara, CA, USA) with the ZORBAX Eclipse XDB-C18 column (4.6 × 150 mm, 5 µM; Agilent Technologies, Santa Clara, CA, USA) and analyzed with a UV photodiode array detector (at 254 nm). The cordycepin was then separated by water and methanol in a ratio of 92:8 (*v/v*), and controlled at a flow rate of 1.0 mL per minute with a retention time of 30 min at 31 C. The cordycepin profiles in the extracts were analyzed in comparison to the cordycepin standard.

### 4.8. The Cytotoxicity Assay of C. Militaris and I. Tenuipes Extracts on CCA Cell Lines

The CCA cell lines had approximately 1 × 10^4^ cells per well and were seeded with a 4-fold serial dilution of the extract at 7.8125–500 µg/mL and incubated for 24 h. Then, the culture media was replaced with the PrestoBlue™ reagent (Invitrogen, Carlsbad, CA, USA) using 100 µL per well. Finally, the plates’ absorbances were measured at the wavelengths of 570 and 600 nm by using a microplate reader that measured the color changes at those levels.

### 4.9. The Killing Assay of NK Cell Enhancing with C. Militaris and I. Tenuipes Extracts

The CCA cell lines that express mCherry (fluorescent cells) were seeded in 96-well plates at approximately 7 × 10^3^ cells per well and incubated for 24 h. Then, the culture media were displaced with the combination of NK cells at an E/T ratio of 1:1 and the extract at a concentration of 31.25 µg/mL. Furthermore, the cells were treated with extract at a concentration of 31.25 µg/mL for 24 h, and then the culture media was replaced with NK cells at an E/T ratio of 1:1. After 24 h, the cells were observed under the fluorescent microscope and the subsequent pictures were analyzed for intensity using ImageJ software. After that, the cells were stained with crystal violet and soaked in 50% ethanol to measure absorbance at 590 nm and to calculate the percentage of remaining cells.

### 4.10. Statistical Analysis

The statistical analysis was performed using the Student’s t-test from GraphPad Prism software, version 9.0.2 (GraphPad Software, Inc., San Diego, CA, USA). The results are shown as the mean ± SEM of at least three independent experiments.

## Figures and Tables

**Table 1:** Cytotoxicity results of cordycepin on CCA cell lines.

Compound	CC_50_ of CCA Cell Lines (µM)		
	CCA-KKU-213A	CCA-KKU-100	CCA-KKU-055
Cordycepin	119.1	>400 *	135.8

* The highest tested concentration.

**Table 2:** Cytotoxicity of the *C. militaris* and *I. tenuipes* extracts on CCA cell lines.

Extract	CC_50_ of CCA Cell Lines (µg/mL)		
	CCA-KKU-213A	CCA-KKU-100	CCA-KKU-055
*Cordyceps militaris*	85.48	122.7	88.88
*Isaria tenuipes*	>500 *	>500 *	>500 *

* The highest tested concentration.

## Data Availability

Not applicable.

## Supplementary Materials

The following are available online. Figure S1: Effect of cordycepin treatment on CCA cell viability, Figure S2: Effect of 100-µM cordycepin on enhancing NK-92 cytotoxicity to kill CCA cells, Figure S3: Effects of cordycepin on modulating CD95, DR4, DR5, MICA/B expression in KKU-055 and KKU-100, Figure S4: The HPLC chromatogram of cordycepin and crude extracts of *C. militaris* and *I. tenuipes*, Figure S5: The effects of cordycepin on modulating IFN-gamma and Granzyme expression in NK-92 cells, Figure S6: The analysis of cell viability by measuring the mean fluorescence intensity (ImageJ software), Table S1: The list of primer sequence.
