# Supplementary material for: Cordycepin Sensitizes Cholangiocarcinoma Cells to Be Killed by Natural Killer-92 (NK-92) Cells
Source: Molecules. 2021 Oct 1;26(19):5973. doi: 10.3390/molecules26195973 (PMC8512070; doi:10.3390/molecules26195973)
Supplement: Supplementary file 1 [file molecules-26-05973-s001.zip › molecules-1357141-supplementary.pdf]

Supplementary materials

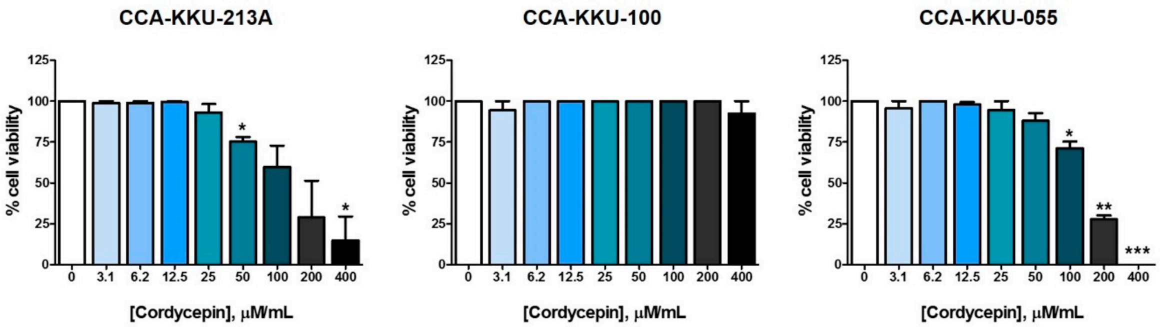

Figure S1: Effect of cordycepin treatment on CCA cell viability. The cell viability was measured after treatment with various concentrations of cordycepin for 24 hours by using cell viability assay compared to that of a no-treatment control.

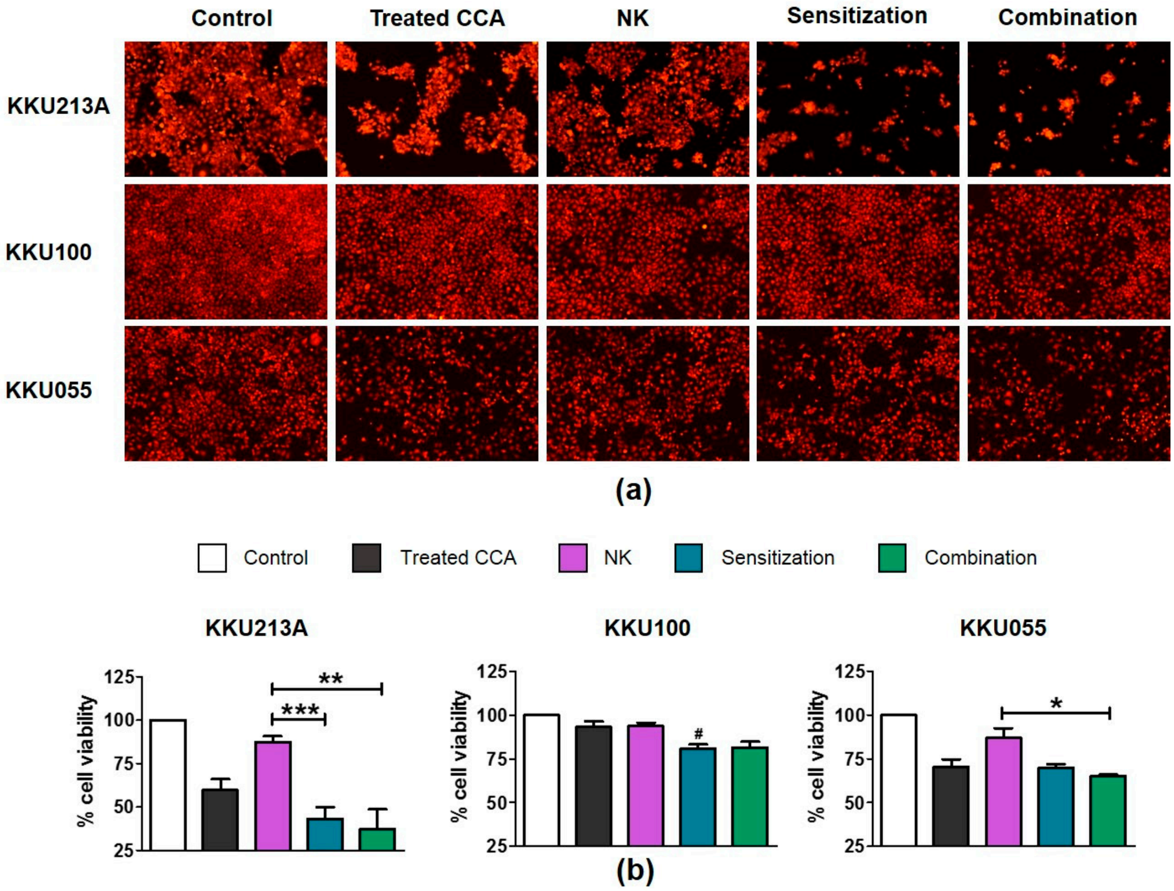

Figure S2: Effect of 100 μM cordycepin on enhancing NK-92 cytotoxicity to kill CCA cells. The effect of cordycepin at the concentration of 100 μM to enhance NK killing ability was determined in KKU-213, KKU-100, and KKU-055 by killing assay. The number of living cells after NK cell co-culture in the absence or presence of cordycepin (under sensitization and combination conditions) was observed under the fluorescence microscope (a). The fluorescence intensity was measured and represented as %cell viability relative to a no-treatment control set as 100% (b).

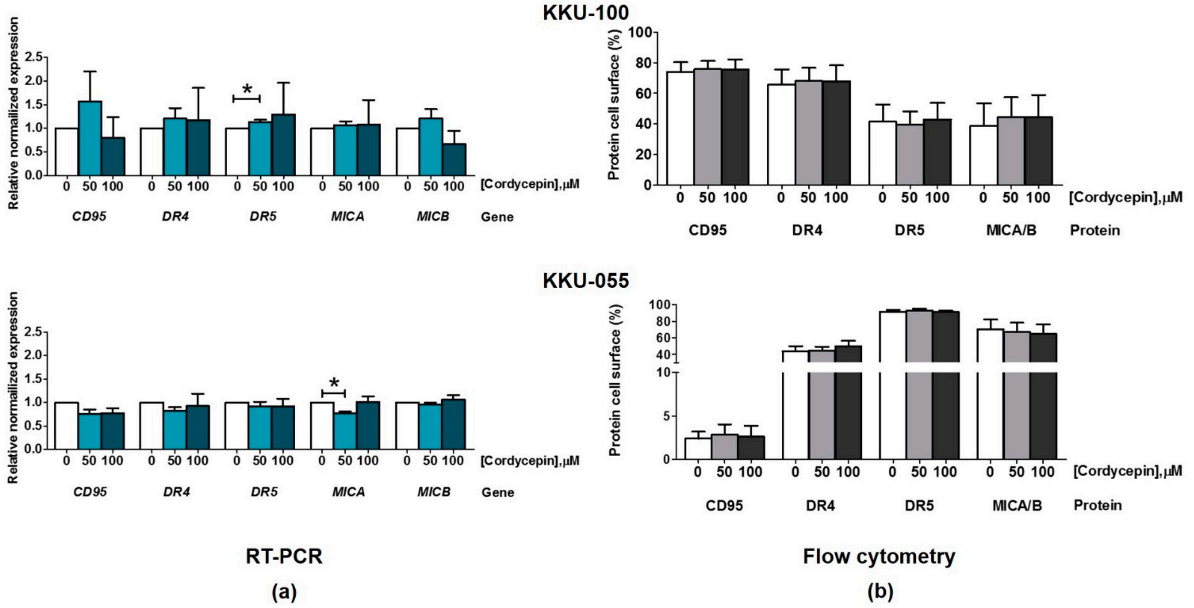

Figure S3: The effects of cordycepin on modulating CD95, DR4, DR5, MICA/B expression on KKU-100 and KKU-055. Cordycepin at the concentrations of 0, 50 and 100 µM were treated in KKU-100 and KKU-055 for 24 hours to determine its effect on expression modulation of CD95, DR4, DR5, MICA/B expression alteration in either mRNA (a) and protein levels (b) by using real-time PCR and flowcytometry respectively.

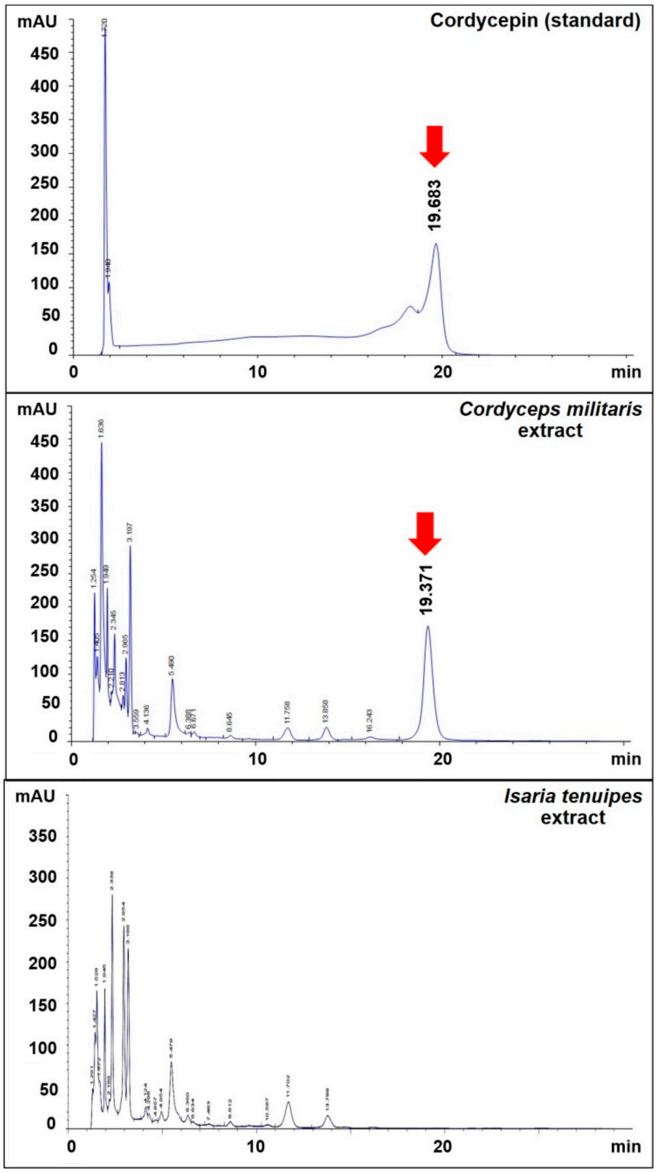

Figure S4: The HPLC chromatogram of cordycepin and crude extracts of *C. militaris* and *I. tenuipes*. The extracts were analyzed by using HPLC under mobile phase; water-methanol (92:8, V/V) with retention time of 19.8 min and detected at a wavelength of 254 nm.

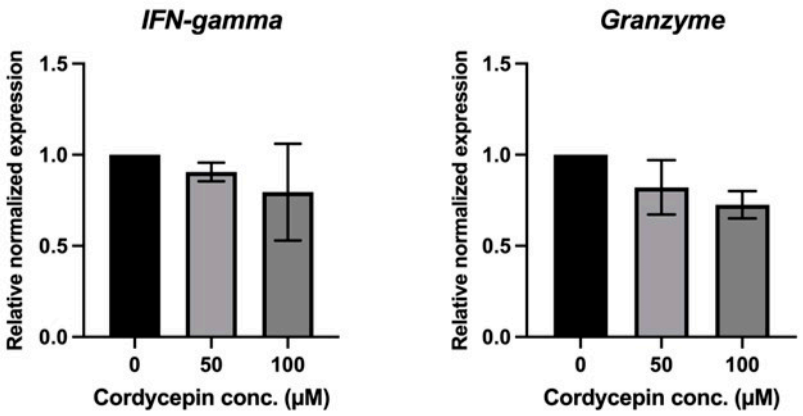

Figure S5: The effects of cordycepin on modulating IFN-gamma and Granzyme expression in NK-92 cells. Cordycepin at the concentrations of 0, 50 and 100 μM were treated in NK-92 cells for 24 hours to determine its effect on expression modulation of IFN-gamma and Granzyme mRNA expression alteration by using real-time PCR.

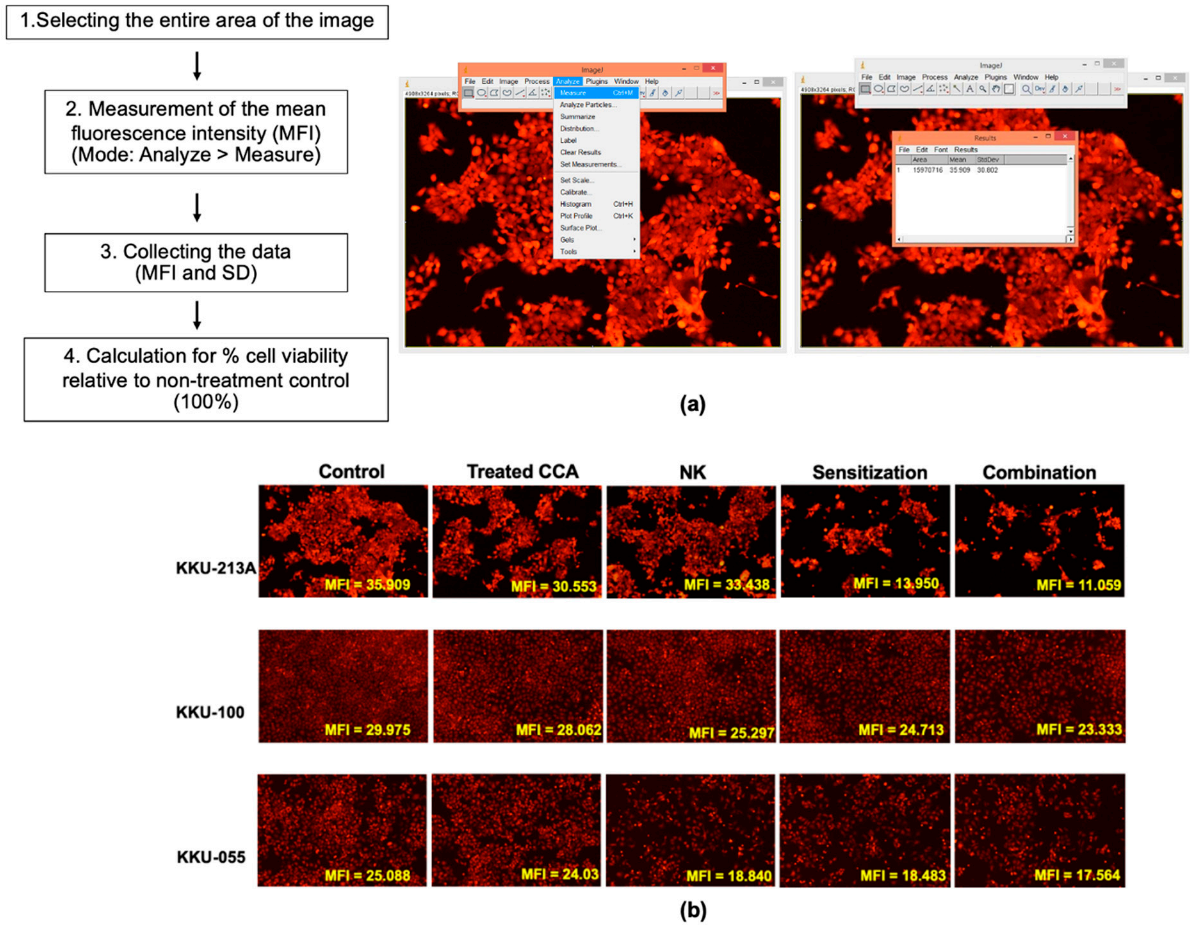

Figure S6: The analysis of cell viability by measuring the mean fluorescence intensity (ImageJ software). The photographs of remaining CCA after 24-hour coculturing of NK and CCA cells was taken and used to analyze the mean fluorescence intensity (MFI) using ImageJ software (a). The representative images of KKU-213A, KKU-100 and KKU-055 representing the MFI values derived from ImageJ software. The MFI values were used to calculate the percentage of cell viability relative to non-treatment control which set as 100% (b).

Table S1: The list of primer sequence

Genes		Sequence (5′-3′)	Reference
CD95	Forward	ATGCTGGGCATCTGGACCCT	Li, J.H., M.S. Kluger, L.A. Madge, L. Zheng, A.L. Bothwell, and J.S. Pober, Interferon-γ augments CD95 (APO-1/Fas) and pro-caspase-8 expression and sensitizes human vascular endothelial cells to CD95-mediated apoptosis. The American Journal of Pathology 2002, 161(4): p. 1485-1495.
	Reverse	CAACATCAGATAAATTTATTGCCA	
DR4	Forward	ACCTTCAAGTTTGTCGTCGTC	Li, J.H., M.S. Kluger, L.A. Madge, L. Zheng, A.L. Bothwell, and J.S. Pober, Interferon-γ augments CD95 (APO-1/Fas) and pro-caspase-8 expression and sensitizes human vascular endothelial cells to CD95-mediated apoptosis. The American Journal of Pathology 2002, 161(4): p. 1485-1495.
	Reverse	AACTCTCCCAAAGGGCTATGT	
DR5	Forward	AAGACCCTTGTGCTCGTTGT	
	Reverse	AGGTGGACACAATCCCTCTG	
MICA	Forward	CACCTGCTACATGGAACACAGC	Rodríguez-Rodero, S., S. González, L. Rodrigo, J.L. Fernández-Morera, J. Martínez-Borra, A. López-Vázquez, and C. López-Larrea, Transcriptional regulation of MICA and MICB: a novel polymorphism in MICB promoter alters transcriptional
	Reverse	TATGGAAAGTCTGTCCGTTGACTCT	
MICB	Forward	CACCTGCTACATGGAACACAGC	

	Reverse	ACATGGAATGTCTGCCAATGATC	regulation by Sp1. European journal of immunology 2007, 37(7): p. 1938-1953.
IFN-gamma	Forward	GATCCA-GCACAAAGCTGTCA	Miyatake, Y., H. Ikeda, A. Ishizu, T. Baba, T. Ichihashi, A. Suzuki, U. Tomaru, M. Kasahara, and T. Yoshiki, Role of neuronal interferon-γ in the development of myelopathy in rats infected with human T-cell leukemia virus type 1. The American journal of pathology 2006, 169(1): p. 189-199.
	Reverse	GACTCCTTT-TCCGCTTCCTT	
**Granzyme**	Forward	TGGGGGACCCAGAGATTAAAA	Morissette, M.C., J. Parent, and J. Milot, Perforin, granzyme B, and FasL expression by peripheral blood T lymphocytes in emphysema. Respiratory research 2007, 8(1): p. 1-9.
	Reverse	TTTCGTCCATAGGAGACAATGC	
